# Radiomics in sporadic microsatellite instable, mismatch repair deficient and Lynch syndrome-associated pancreatic ductal adenocarcinoma: a pilot study

**DOI:** 10.3389/fonc.2025.1584167

**Published:** 2026-02-20

**Authors:** Ellis L. Eikenboom, Joséphine Magnin, Remo Alessandris, Natally Horvat, Mithat Gonen, William R. Jarnagin, Jeffrey Drebin, Michael I. D’Angelica, T. Peter Kingham, Vinod P. Balachandran, Kevin C. Soares, Anja Wagner, Manon C. W. Spaander, Jayasree Chakraborty, Alice C. Wei

**Affiliations:** 1 Hepatopancreatobiliary Service, Department of Surgery, Memorial Sloan Kettering Cancer Center, New York, NY, United States; 2 Department of Clinical Genetics, Erasmus Medical Center Cancer Institute, University Medical Center Rotterdam, Rotterdam, Netherlands; 3 Department of Gastroenterology and Hepatology, Erasmus Medical Center Cancer Institute, University Medical Center Rotterdam, Rotterdam, Netherlands; 4 Department of Digestive Surgical Oncology, University Hospital of Dijon, INSERM 1432, University of Bourgogne, Dijon, France; 5 Department of Radiology, Memorial Sloan Kettering Cancer Center, New York, NY, United States; 6 Department of Epidemiology and Biostatistics, Memorial Sloan Kettering Cancer Center, New York, NY, United States

**Keywords:** pancreas ductal adenocarcinoma, Lynch syndrome, MSI-H, MMRd, radiomic analysis

## Abstract

**Introduction:**

Pancreatic cancer, mostly presenting as pancreatic ductal adenocarcinoma (PDAC), has a poor prognosis. The microsatellite-instable (MSI-H)/mismatch repair deficient (MMRd) subtype, however, is more susceptible to immune therapy and is expected to have a better prognosis. Presently, MSI-testing is not routinely performed on PDAC. We assessed whether quantitative imaging features (radiomics) of pretreatment computed tomography (CT) scans could diagnose MSI-H/MMRd.

**Methods:**

For this pilot study, we analyzed CT-scans of treatment-naïve sporadic or Lynch syndrome (LS)-associated MSI-H or MMRd PDACs, diagnosed or treated in a single center from 2007 to August 2022. CT-scans of resected MSI-stable, MMR proficient, non-LS PDACs formed a control group, after random selection in 1:4 ratio. Upon CT-scan segmentation, 254 well-defined radiomic features were extracted from pancreas and tumor regions. The predictability of the features was assessed within a repeated stratified 3-fold cross-validation framework by designing three models using random forest classifier, with the most discriminating features selected through the minimum redundancy maximum relevance method from three feature sets: tumor radiomics, pancreas radiomics, and combined tumor + pancreas radiomics. Performance was evaluated by area under receiver operating curve (AUC), sensitivity, specificity, positive and negative predictive value.

**Results:**

Overall, 95 patients were included: 19 patients with MSI-H/MMRd/LS (36.8% female; median age at diagnosis 72 [IQR 60–77 years]) and 76 matched controls with PDAC (53.9% female; median age at diagnosis 66 [IQR 57–74 years]). Median year when CT-scan was done was 2017 and 2018, respectively. The model using radiomic features from the pancreatic tumor reflecting MSI-H/MMRd, had an area under receiver operating curve (AUC) of 0.73. The performance of the model was improved by also incorporating radiomic features from pancreas texture (AUC of combined model 0.83 sensitivity 84%, specificity 78%, negative predictive value 95%).

**Conclusions:**

This pilot study suggests that radiomic features could be used to determine MSI/MMRd status in CT-scans of PDAC, but needs further independent multi-site validation in larger cohorts. Routine application of radiomics to determine MSI-status might be of interest in clinical practice to select patients who could benefit from immune therapy.

## Introduction

Pancreatic ductal adenocarcinoma (PDAC) is the most common type of pancreatic cancer, accounting for approximately 90% of all cases (1). It arises from the cells lining the ducts of the pancreas and is typically diagnosed in an advanced stage, when it has already metastasized to other organs (2). PDAC is notoriously difficult to manage, with a low overall survival rate and limited treatment options (1). Hence, early stage diagnosis is crucial to improve outcomes (3). The lifetime risk of developing PDAC is generally low (4) except for individuals with germline mutations in specific cancer-related genes (5). An example for this is the Lynch Syndrome (LS), caused by a germline pathogenic variant (PV) in one of the alleles of DNA mismatch repair (MMR) genes *MLH1*, *MSH2*, *MSH6*, *PMS2*, or the 3’ end of *EpCAM* (6–10). This increases chances of a second, sporadic PV in the wild-type MMR allele resulting in hampered DNA MMR (mismatch repair deficiency, MMRd)and can lead to accumulation of multiple PVs, ultimately resulting in cancer. LS carriers are at particularly high risk of developing colorectal (CRC) and endometrial cancer (EC), and are therefore advised regular colonoscopies and gynecological surveillance visits. Because DNA MMR is part of housekeeping functions in all replicating cells, carriers also have an increased risk of other tumors, including PDAC (11). Although this risk is higher than the general population (estimated cumulative incidence of maximally 6.2 in *MLH1* carriers compared to 0.66 in the general population up to age 75), pancreas surveillance is not globally recommended in all Lynch carriers (2, 11).

A key feature of LS-related cancers and lesions due to sporadic DNA MMR PVs is microsatellite instability (MSI-H). This refers to length variations in short, repetitive DNA sequences scattered throughout the genome that arise when the mismatch repair (MMR) system fails to correct replication errors. The accumulation of these errors can be detected by various genetic tests (12). MSI-H tumors, specifically CRCs, are known to have a favorable prognosis compared to their MSI-stable (MSS) counterparts (13). Notably, the prognosis of MSI-H PDACs also seems to be slightly better, although literature on this topic is scarce (14). Despite the generally more favorable prognosis in MSI-H/MMRd tumors, routine testing of these features has been predominantly endorsed for CRC and EC, not for other tumor types (15–17). Although the absolute numbers of MSI-H non-CRC and non-EC tumors are substantial, systematically testing for MSI or MMR status is probably not cost-effective as the relative numbers per tumor type are low, e.g. 1-2% for PDAC (18, 19). However, one can imagine that an alternative approach such as radiomics on routine CT-scans could in theory, if automated, determine MSI-status in a cheaper manner.

Radiomics is the quantitative analysis of radiographic images with a large number of features and has showed promising performance and is increasingly used in detection, diagnosis, prognosis, and staging of different cancers, including pancreatic cancers (20–29). In brief, radiomic features capture intensity, shape, size, and/or texture, of an organ or tumor that reflect underlying pathophysiology (30). Specifically, texture analysis provides a measure of intratumoral heterogeneity, which can assess tumor aggressiveness and was shown to be predictive of tumor grade, response to therapy, genotype, and stromal content (23, 27, 28, 31–33). Radiomics has also shown promising performance in detecting MSI-H status for several cancers, such as colorectal cancer and colorectal liver metastasis, but has not been explored for PDAC (25, 34–36). This preliminary, proof-of-principle study aimed to assess if radiomics of CT-scans can identify MSI-H/MMRd status in PDAC, paving the way for more accurate and personalized prognosis and treatment options. Specifically, as tumors with hereditary or sporadically developed PVs in DNA MMR genes are usually more susceptible to immunotherapeutic agents, even in the metastatic setting (37–40).

## Methods

### Identifying study participants

To identify patients with MSI-H, MMRd, or LS, we queried all PDAC tumors resected at MSK in patients diagnosed with LS or that were sequenced through August 10^th^ 2022. All included patients consented to sequencing of the tumor by MSK-IMPACT (IRB 12-245), which is standard of care at the institution since 2014 (41). Currently, MSK-IMPACT consists of 468 cancer-related genes, including DNA MMR genes (*MLH1*, *MSH2*, *MSH6*, *PMS2*, or *EpCAM*) and detection of MSI-H. Briefly, blood cells are used as a reference to call for somatic variants in the tumor. Additionally, some patients also consent to germline sequencing for cancer-related genes (42). For some patients, LS diagnostics were performed in another center and PDAC sequencing was not performed. Therefore, presence of germline PVs was also determined from patient files. All sequencing data were anonymously and prospectively stored in C-bioportal. Baseline characteristics including clinical, pathologic, and outcome data were retrieved from corresponding patients’ medical records.

Pathogenicity of both sporadic and germline DNA MMR variants was retrieved via the sequencing report or manually assessed via NCBI ClinVar (for sequencing outcomes reported prior to January 1^st^ 2022) (43). In case of a likely pathogenic or pathogenic variant, PDACs were classified as MMRd and patients were included for further review. Also, in case of MSI-H or indeterminate MSI status, as assessed via MSISensor score, patient records were further assessed (44, 45). Similarly, sporadic DNA MMR protein status was assessed by immunohistochemistry (IHC). In case of absence of at least one DNA MMR protein, tumors were considered MMRd. We considered MMRd PDACs to be MSI-H as well.

Patients with molecularly confirmed PDAC and that were MSI-H-positive, had aberrant DNA MMR IHC, or were known carriers of LS, and had available treatment-naïve CT-scans in the institutional picture archiving and communication system (Centricity PACS, GE Healthcare), were included in our study (N=19). The control group (N=76) was randomly selected and comprised of surgically resected patients with PDAC, not having MSI-H, MMRd, or a prior LS diagnosis at MSK between 2011 and 2021, and had available treatment-naïve portal venous phase contrast CT-scans.

### Quantitative CT image analysis

For all patients, contrast-enhanced CT scans were acquired in the context of standard-of-care pretreatment diagnosis. Upon administration of 150 mL of iodinated contrast (Omnipaque 300, GE Healthcare) at 4.0 mL/s, CT-scans were made by a multidetector CT (Lightspeed 16 and VCT, GE Healthcare). Both pancreatic parenchymal phase and portal venous phase were scanned, with axial slice intervals ranging from 1.25 to 5.00 mm and parameters as described previously. For the analysis in the proposed study, portal venous phase CT scans were used (33). The study design was approved by the MSK institutional review board (IRB 21-224).

Radiomics analyses were performed with all available treatment-naïve CT-scans. Both the pancreatic tumor and the pancreas were manually segmented by a trained physician (J.M.), using Terarecon^®^ software (Terarecon Inc., Foster City, California, USA). Segmentations were checked by a clinical radiologist (N.H) experienced in pancreas segmentations. 254 well-defined radiomic features were then extracted from the segmented pancreas and pancreatic tumor volume, described previously by our team. (25, 46).

Among these features, gray-level-co-occurrence matrices (GLCM), intensity-histograms, run-length matrices (RLM), fractal dimension (FD), and local binary patterns (LBP) quantify intensity patterns. Angle co-occurrence matrices (ACM) quantify edge patterns. All features were extracted from each axial CT slice and averaged to a single value for each feature.

### Statistical analyses

Baseline characteristics were assessed for both groups. Data were presented as medians with interquartile range (IQR) for continuous variables and as numbers with percentages for categorical data. Mann-Whitney U tests and χ^2^ (Fisher’s exact) tests were used to determine statistical differences between MSI-H PDACs and the control group.

P-values of <0.05 were considered statistically significant. Analyses were performed in SPSS (version 28.0.1.0).

Association between each radiomic feature with MSI-status was assessed by a Wilcoxon-rank-sum-test. To determine predictability of the radiomic features, three models predicting MSI status were designed with different classifiers using radiomic features: first, a prediction model using features extracted from the tumor (TumRad); second, a prediction model using features extracted from the surrounding pancreas (PancRad); and third, a prediction model combining features from both tumor and pancreas tissue (IntRad) and evaluated with repeated stratified 3-fold cross-validation technique (100 runs) to avoid overfitting. Specifically, feature selection and model building were conducted independently within each fold using training data only and were evaluated on the corresponding test data (47). Due to a small sample size in the MSI-H group, we did not employ an independent hold out set in this study (47, 48).

The first two prediction models (TumRad and PancRad) were designed using logistic regression, support vector machine (SVM), random forest, and XGBoost classifier, after selecting most discriminating features using the minimum-redundancy maximum-relevance (mRMR) method followed by a forward selection method. The mRMR method selected features that highly correlated with MSI-status, but had a low correlation with other features. To design the combined model (IntRad), the probability scores obtained from TumRad and PancRad were integrated using logistic regression-based classifier. This model was also evaluated using stratified 3-fold cross-validation in the same repeated manner. The output of each classifier provides a risk score between 0 and 1, corresponding to the level of confidence that a patient would achieve a response following the conclusion of therapy.

**Figure 1:**
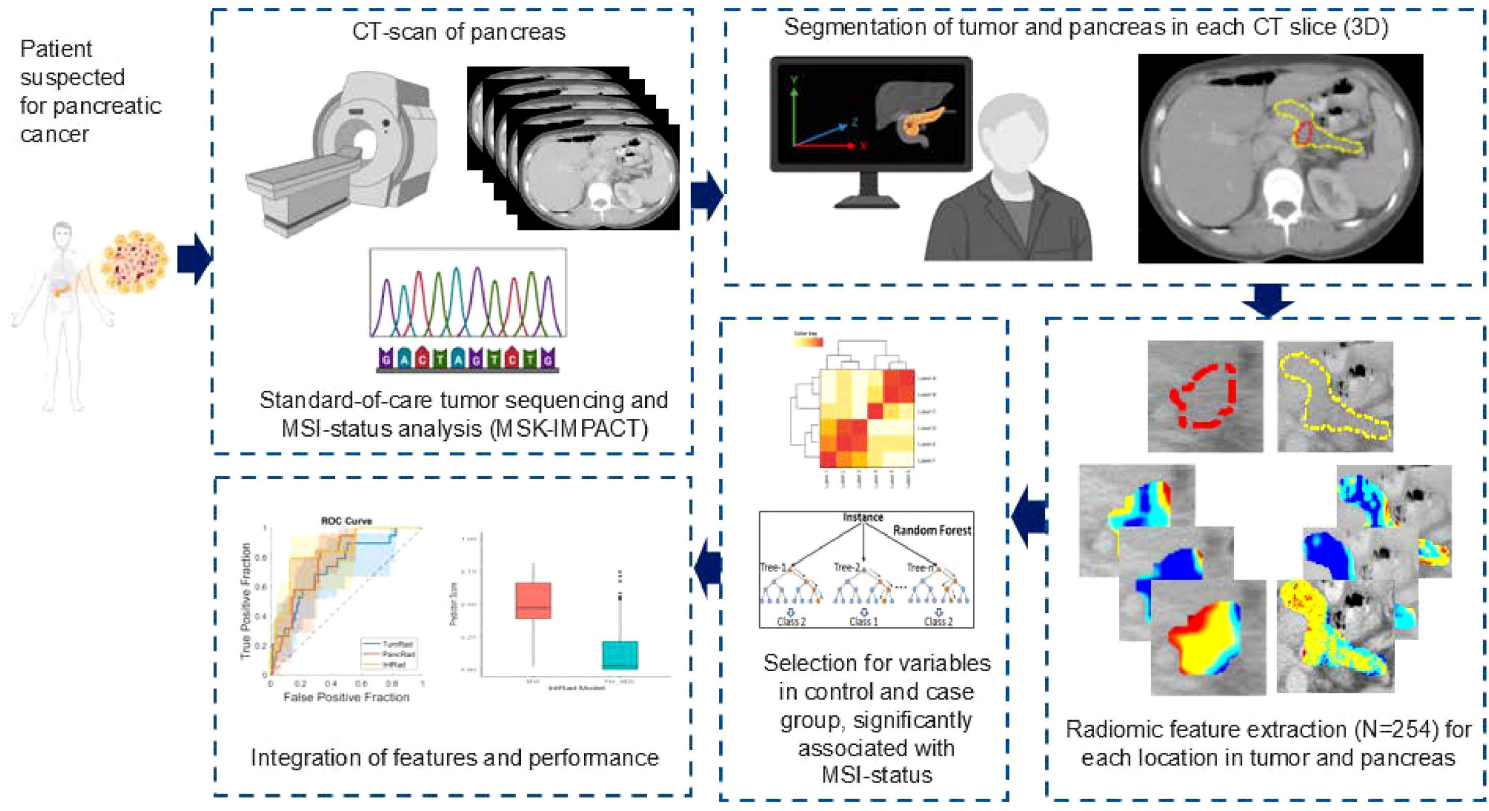
Schematic overview of radiomic analyses for the current study.

Performance of these multivariate analysis were assessed by the Area Under the receiver operating characteristic Curve (area under ROC curve, AUC), sensitivity, specificity, positive predictive value (PPV), and negative predictive value (NPV), with corresponding 95% confidence intervals (CI). The sensitivity, specificity, PPV, and NPV were computed dichotomizing the radiomic-based risk score using a threshold that optimizes both sensitivity and specificity (Youden index). ROC and decision curve analyses (DCA) were used to visualize diagnostic performance and clinical utility, respectively. Analyses were performed with MATLABR2017a (The Mathworks Inc.) software. See **Figure 1** for a schematic overview of the proposed approach.

## Results

### Patient characteristics

Between May 2007 and August 2023, 19 MSI-H PDACs met inclusion criteria and were included in our analysis (see **Supplementary Table 1** for an overview of patient characteristics). Among these, seven patients had LS, with one carrying a PV in *MLH1*, one in *MSH2*, one in *MSH6*, and four in *PMS2*. The other twelve had developed sporadic MSI-H PDAC (four *MLH1*, one *MSH2*, seven *MSH6*; **Table 1**). Of the 19 included patients, nine had surgery for their PDAC. A total of 76 surgically resected and previously segmented PDACs were subsequently included as controls. Median age at surgery was 72 years for the MSI-H group (interquartile range (IQR) 60–77 years), 65 years for the control group (IQR 57–74 years; p=0.141). CT-scans of pancreatic cancers of both groups were mostly obtained after 2016 and the average year of CT-scan did not differ significantly between groups (median year of performance of CT scan 2017 for cases and 2018 for controls, p=0.781). Neoadjuvant therapy was provided significantly more often in the case group, but after the CT-scan was obtained (MSI-H 73.7% versus control group 21.7%; p<0.001). Additional clinical and pathological characteristics of both groups can be found in **Table 1**.

**Table 1:** Baseline characteristics of MSI-H and control pancreatic ductal adenocarcinomas.

Characteristics	MSI-H^*^ (n=19)	Controls (n=76)	P-value
**Gender, female, N (%)**	7 (36.8)	41 (53.9)	.208
**Age at diagnosis, median (IQR)**	72 (60-77)	66 (57-74)	.226
**Year of CT, median (range)**	2017 (2007-2021)	2018 (2010-2021)	.836
**MMR gene involved, N (with N of whom caused by Lynch syndrome)** ***MLH1*** *** MSH2*** *** MSH6*** *** PMS2***	5 (1) 2 (1) 8 (1) 4 (4)	N/A	N/A
**Neoadjuvant treatment, N (%)** ** No** Yes^*^	5 (26.3) 14 (73.7)	57 (75.0) 19 (25.0)	<.001
**BMI, median (IQR)**	25 (24-27)	27 (24-30)	.265
**History of alcohol abuse, N (%)**	3 (15.8)	5 (6.6)	.162
**Smoking status, N (%)** ** Never** ** Current/former** ** Unknown**	10 (52.6) 8 (42.1) 1 (5.3)	39 (51.3) 36 (47.4) 1 (1.3)	.800

N/A, not applicable; ^*^Including chemotherapy, radiotherapy, and immune therapy in MSI-H group; including chemotherapy and radiotherapy in control group. ^*^In total, nine of the 19 included MSI-H PDAC patients had surgery for their PDAC.

### Prediction of MSI-status

Following segmentation and radiomic feature extraction from pancreatic cancers and normal pancreas tissue, 62 features from tumor tissue and 86 features from pancreas tissue were found to be significantly associated with MSI status (see **Supplementary Table 2**). With mRMR-based feature selection from the tumor region, two fractal dimension (FD31 and FD9) and two run-length matrices (RLM11 and RLM9)-based features were most frequently selected, appearing more than 70% of the time. Similarly, three fractal dimensions (FD31, FD37, FD48) and one local binary pattern (LBP72) were the most frequently selected features from normal pancreas tissue.

**Figure 2:**
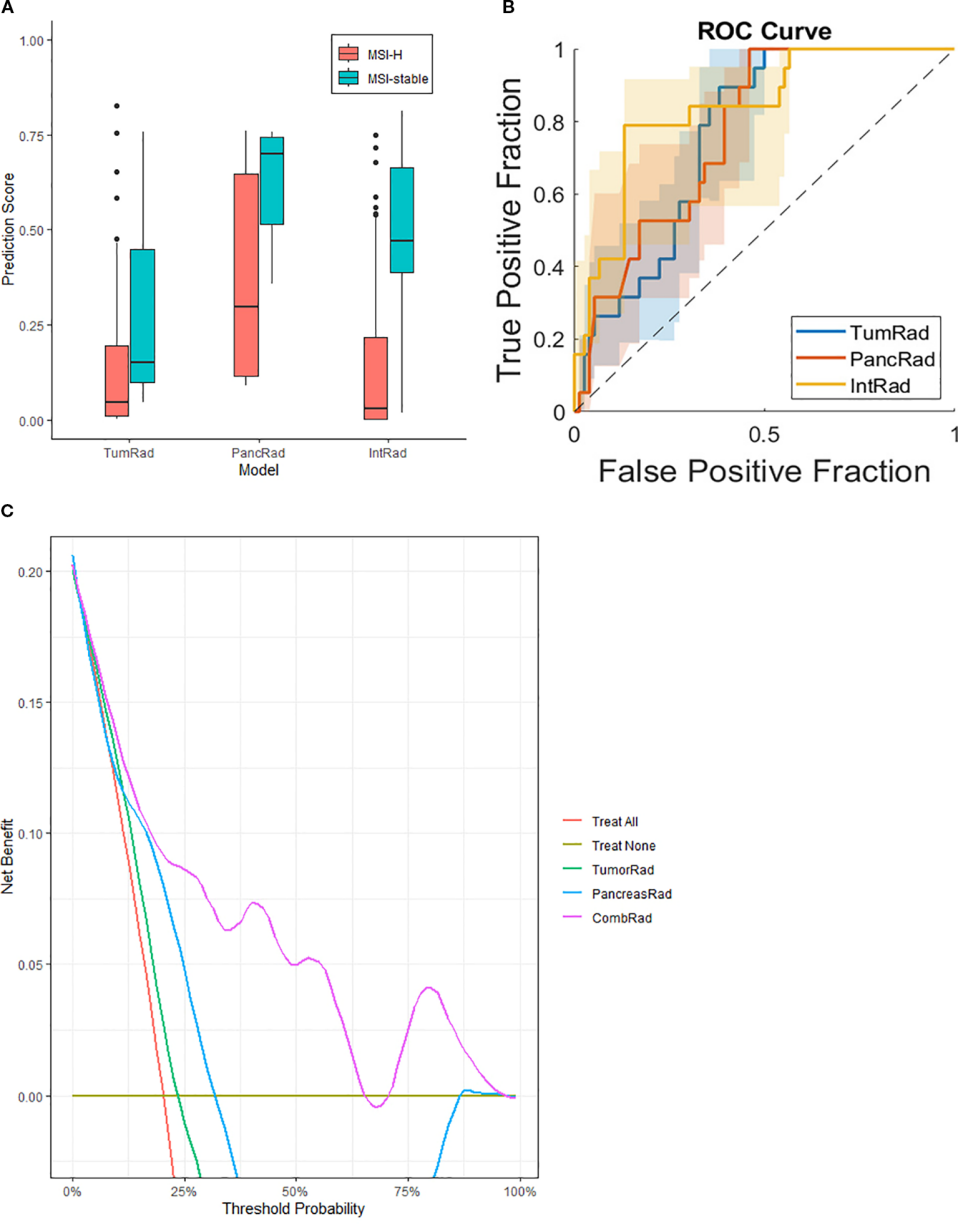
Quantitative image analysis, predicted MSI-status in PDAC. Box plots, provided for the best classification for each region, of **(A)** Prediction scores of TumRad model, with a discrimination slope of 0.20; PancRad model, with a slope of 0.25; andIntRad model, with a slope of 0.32. **(B)** Area Under the receiver operating characteristic Curve (AUC) of the two separate models (blue and red lines) and both models combined (yellow line). The discrimination slope is calculated as the difference between the mean predicted scores of MSI-H and MSI-stable groups. **(C)** Decision Curve Analysis (DCA) curve for independent and combined prediction models.

The TumRad model showed an AUC of 0.76, sensitivity of 77%, and NPV of 93% (**Figures 2A, B**, **Table 2**) with XGBoost classifier. The PancRad model had comparable performance metrics with an AUC of 0.77, sensitivity of 74%, and an NPV of 93% (**Figures 2A, B**, **Table 2**).

**Table 2:** Performance of predicted MSI-status in PDAC.

Features	Classifiers	AUC	Sensitivity	Specificity	PPV	NPV
Pancreas tumor radiomics (TumRad)	Logistic Regression	0.67 (0.56-0.77)	68% (48%-89%)	66% (55%-77%)	33% (19%-48%)	89% (81%-97%)
	SVM	0.72 (0.62-0.86)	74% (54%-94%)	65% (54%-75%)	34% (20%-49%)	91% (83%-99%)
	XGBoost	0.76 (0.65-0.85)	77% (61%-97%)	67% (57%-78%)	38% (22%-53%)	93% (86%-100%)
	Random Forest	0.72 (0.62-0.86)	68% (48%-89%)	72% (62%-82%)	38% (22%-55%)	90% (83%-98%)
Pancreas radiomics (PancRad)	Logistic Regression	0.74 (0.64-0.84)	74% (54%-94%)	66% (55%-77%)	35% (20%-50%)	91% (83%-99%)
	SVM	0.76 (0.65-0.85)	74% (54%-93%)	70% (59%-80%)	38% (22%-54%)	91% (84%-99%)
	XGBoost	0.67 (0.56-0.77)	68% (48%-89%)	67% (55%-77%)	36% (20%-50%)	90% (83%-98%)
	Random Forest	0.77 (0.69-0.88)	74% (54%-94%)	79% (70%-88%)	47% (29%-65%)	93% (86%-99%)
Combined features (IntRad)	Logistic Regression	0.79 (0.70-0.90)	74% (54%-94%)	70% (59%-80%)	38% (22%-54%)	91% (83%-99%)
	SVM	0.81 (0.71-0.91)	89% (76%-100%)	67% (57%-78%)	41% (26%-55%)	96% (91%-100%)
	XGBoost	0.81 (0.71-0.92)	79% (61%-97%)	78% (68%-87%)	47% (30%-64%)	94% (88%-100%)
	Random Forest	0.83 (0.74-0.94)	84% (68%-100%)	78% (68%-87%)	49% (31%-66%)	95% (90%-100%)

The 95% confidence intervals are indicated in parentheses.

AUC = Area Under the receiver operating characteristic Curve; NPV = negative predictive value; PPV = positive predictive value; SVM = Support Vector Machine; XGBoost = eXtreme Gradient Boosting.

**Figure 3:**
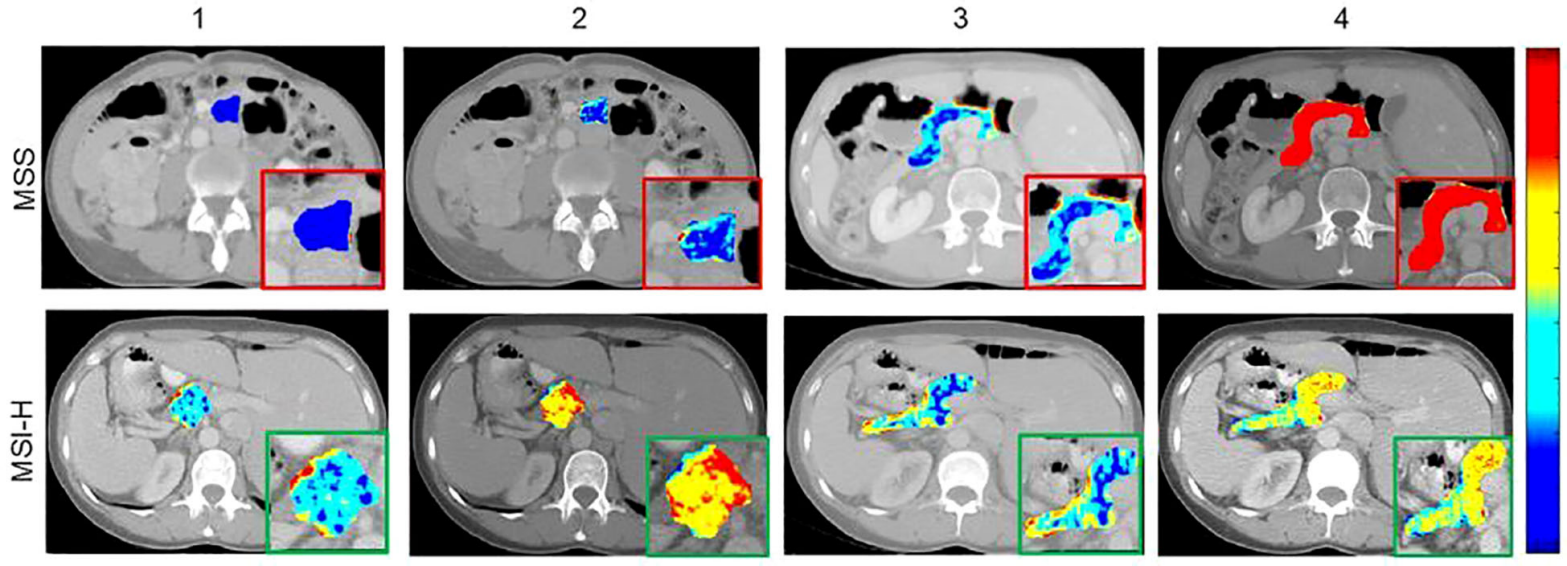
Radiomic feature maps of pancreatic tumor and pancreas for microsatellite stable (MSS) and MSI-H patients. RLM- and FD-based features from tumors (columns 1 and 2) and FD-based features from pancreas (columns 3 and 4) illustrated higher heterogeneity. Red indicates higher values of radiomic features and blue indicate lower values.

Subsequently, both models were integrated into a combined model (IntRad), which showed improved performance, with an AUC of 0.83, sensitivity of 84%, specificity of 78%, and an NPV of 95% (**Figures 2A, B**, **Table 2**). Other performance metrics of these models are provided in **Table 2**. Decision Curve Analysis (DCA) further demonstrated that the combined radiomics model provided the highest net benefit across a clinically relevant threshold range (10–70%), outperforming both single-region models (TumorRad, PancreasRad) and default treatment strategies (treat-all, treat-none; **Figure 2C**). Examples of MSI-H and MSI-stable CT scans with selected radiomic features can be found in **Figure 3**.

## Discussion

Although not routinely performed in PDAC, routine assessment of MSI-status is relevant to distinguish those tumors that could potentially benefit from immune therapy. Therefore, we explored the role of radiomics in predicting MSI-status in pre-treatment-CT-scans of PDACs in this preliminary proof-of-concept study with 19 MSI-H and 76 controls,. Various texture features of tumor and healthy pancreas tissue were used to build a combined model. An adequate discriminatory ability (AUC of 0.84, specificity of 87%, NPV of 94%). However, this model needs further validation in a larger cohort of patients.

### Advantages of radiomic analyses

Recently, multiple studies of radiomics analyses of PDACs have underscored its promising applications in both molecular research and clinical practice (28, 33, 49–53). Radiomics analyses do not only generate additional information about tumor biology – they outperform the prognostic value of clinical data alone (54) and present advantages over commonly used alternatives, such as tumor DNA-sequencing. Radiomics analyses do not require tissue, which is generally obtained through invasive procedures such as biopsy or surgery. This holds special value for the significant percentage of patients who are diagnosed at a late and unresectable stage (1). Additionally, compared to biopsies that are captured randomly and could therefore miss tumor heterogeneity, radiomics assess the entirety of pancreas and tumor texture (55). Recent studies indicate that radiomics can even predict PDAC driver gene status (33, 52).

### Applications for radiomics in MSI-H PDACs

Due to their different genomic constitution, MSI-H tumors are expected to have a different prognosis and an altered response to neoadjuvant treatment (56); this was also shown for PDACs specifically (14, 57). A recent study found improved survival of patients with MSI-H tumors who were treated with immune therapy, although its effect was contradictory for included PDACs (39). Determining the MSI-status of PDAC prior to resection therefore is relevant to the stratification of patients to different neoadjuvant or adjuvant treatment regimens (38, 39, 58). Similarly, previous research showed that radiomic analyses, if properly validated, could also be used to more accurately predict survival in resectable PDACs (51, 53, 59). Overall, there is growing evidence, including this study, that radiomics is a reliable, non-invasive, relatively low-cost tool for predicting genomics and prognosis of PDAC, as well as informing treatment selection.

### Limitations

Our study has several limitations. First, we were limited by the small number of MSI-H PDACs. Although we searched Memorial Sloan Kettering’s large volume of consecutive patients with PDAC from 2007-2022, due to low prevalence of MSI-H PDAC (5, 60, 61), we could not validate the model in an independent dataset. Instead, we only performed cross-validation. However, to confirm the predictability of the radiomic features, we repeated the stratified cross-validation for 100 times and provided the average performance over all the runs. Future studies are needed to validate these features, preferably using larger and more heterogeneous groups of PDAC patients (multicenter study). Second, as a consequence of MSI-H PDAC rarity, we could not select for quality parameters in CT-scans. The oldest included CT-scan dated from 2007. In future studies, findings should be validated in recent CT-scans of independent PDAC patients. Third, the control group differed significantly from the case group with respect to performance of PDAC surgery as we used a previously segmented surgical cohort of PDAC and included only MSI-H, MMRd, and LS-associated PDACs as cases, regardless of having had surgery. Selecting for surgically resected MSI-H PDACs only would leave us with a very small case group, and so far, resectability is not known to be directly associated with MSI-status. We avoided matching controls to our case group as we believed this would introduce bias into the model given our small case cohort. Similarly, results of a matched analysis are only generalizable to the records that can be matched. Instead, we therefore chose to randomly select controls instead and to transparently report possible differences between groups (such as the significant difference between both groups with regards to neoadjuvant therapy). As we only used pre-treatment CT-scans, we believe these features would not have significantly affected our model and we therefore chose not to include these features in this pilot study. This was confirmed in subgroup analyses evaluating the performance of the models separately for appliance of neoadjuvant therapy and TNM stage (**Supplementary Table 3**).

Fourth, the proposed radiomic analysis relied on labor-intensive manual segmentation. While pancreas segmentation can be automated, tumor segmentation still requires manual effort due to the lack of an accurate automated tool for this task. Pancreatic tumors are notoriously difficult to segment due to their vague boundaries leading to inter-observer variability during segmentation (48). Regardless, recent studies were able to differentiate between patients with healthy pancreas tissue and patients with PDAC, as pancreas tissue containing PDACs displayed lower image intensity and higher heterogeneity, and as larger PDACs probably affected the shape of the healthy pancreas (37, 38). However currently, no accurate auto-segmentation tools are available for PDAC. Therefore, radiomic studies in our center are still performed by manual segmentation. To check the impact of inter-reader variability, a small subset of tumors (N=10) was segmented by another reader. Here, substantial inter-reader agreement (kappa = 0.74) was found. In future studies, we will also quantitatively assess the impact of inter-reader variability on the radiomic features in a larger cohort of patients.

### Conclusion and future directions

In conclusion, to our knowledge, this is the first study to assess whether radiomic analyses could aid in the determination of MSI-status in PDAC. In this pilot study, we built a radiomics model based on both pancreas tumor and tissue texture that showed potentiality of radiomic features in predicting MSI-status in a small group of PDACs from a single center. These findings should be validated in an independent dataset consisting of a larger, heterogeneous patient population. To obtain such a cohort, preferably an international, multicenter should be carried out, in centers using comparable CT-scan protocols. This cohort should also allow robust subgroup analyses to assess the possible impact of clinicodemographic factors. To explore model validation, specifically those centers that routinely assess MSI or MMRd status in PDAC should be invited to participate. If validated, application of routine radiomics in PDAC CT-scans could aid personalized treatment decisions.

## Data Availability

The raw data supporting the findings of this study will be made available by the authors upon request and in accordance with institutional guidelines. Requests for access should be directed to the corresponding authors.
