# Supplementary material for: Radiomics in sporadic microsatellite instable, mismatch repair deficient and Lynch syndrome-associated pancreatic ductal adenocarcinoma: a pilot study
Source: Front Oncol. 2026 Feb 20;15:1584167. doi: 10.3389/fonc.2025.1584167 (PMC12962943; doi:10.3389/fonc.2025.1584167)
Supplement: Supplementary file 1 [file Table1.docx]

**Supplementary Table 1** Patient characteristics of included patients with pancreatic ductal adenocarcinoma being MSI-H, mismatch repair deficiency, or Lynch syndrome-associated.

| ID | Age at PDAC surgery, years | Sex | Lynch syndrome or sporadic | Gene involved* | MSI and DNA MMR IHC | TMB |
| --- | --- | --- | --- | --- | --- | --- |
| M002 | 45 | Female | Lynch syndrome | *MLH1* | MSI-H | 21 |
| M004 | 63 | Male | Lynch syndrome | *PMS2* | MSI-H | 85.6 |
| M005 | 74 | Male | Lynch syndrome | *PMS2* | MSS | 5.9 |
| M006 | 64 | Female | Sporadic | *MLH1* | MSS | 7 |
| M008 | 85 | Male | Sporadic | *MSH2* | MSI-H | 47 |
| M009 | 76 | Female | Lynch syndrome | *PMS2* | MSS | 5 |
| M010 | 54 | Male | Sporadic | *MLH1* | MSS | 3 |
| M012 | 77 | Female | Sporadic | *MSH6* | MSI-H, MMRd | 69.3 |
| M013 | 76 | Female | Sporadic | *MSH6* | MSS | 4 |
| M015 | 78 | Female | Sporadic | *MSH6* | MSI-H | 42 |
| M016 | 75 | Male | Sporadic | *MSH6* | MSS | 6 |
| M017 | 72 | Male | Lynch syndrome | *MSH6* | MSI-H | 44 |
| M018 | 83 | Male | Sporadic | *MSH6* | MSS | 3 |
| M019 | 60 | Male | Lynch syndrome | *PMS2* | MSS | 0 |
| M021 | 77 | Male | Sporadic | *MSH6^+^* | MSS, IHC equivocal staining | 36.3 |
| M022 | 72 | Male | Sporadic | *MLH1* | MSS | 27.2 |
| M023 | 49 | Male | Sporadic | *MSH6* | MSS | 1.6 |
| M025 | 53 | Male | Sporadic | *MLH1* | MSI-H | N/A |
| M026 | 66 | Female | Lynch syndrome | *MSH2* | N/A | N/A |

MSI-H = microsatellite instability-high; MMRd = mismatch repair deficient; IHC= mismatch repair immunohistochemistry; TMB = tumor mutational burden; MSS= microsatellite stable; N/A = not tested.

*Pathogenic variants or likely pathogenic variants in MMR genes only. ^+^ Pathogenicity of variant unknown, but assumed pathogenic as PDAC was found to be MSI-H.
