# Supplementary material for: Radiomics in sporadic microsatellite instable, mismatch repair deficient and Lynch syndrome-associated pancreatic ductal adenocarcinoma: a pilot study
Source: Front Oncol. 2026 Feb 20;15:1584167. doi: 10.3389/fonc.2025.1584167 (PMC12962943; doi:10.3389/fonc.2025.1584167)
Supplement: Supplementary file 3 [file Table3.docx]

**Supplementary Table 3** Subgroup analyses for effect of neoadjuvant therapy and tumor staging on combined pancreas radiomics.

|  | Sensitivity | Specificity |
| --- | --- | --- |
| Neoadjuvant therapy^∆^ |  |  |
| Yes | 79%  (57%-100%) | 89%  (76%-100%) |
| No | 80%  (45-100%) | 86%  (77%-95%) |
| TNM Stage^◦^ | | |
| Stage I | 67%  (13%-100%) | 93%  (81%-100%) |
| Stage II | 67%  (33%-84%) | 85%  (41%-97%) |

^∆^ In the case group, 14 out of 19 patients received neoadjuvant treatment, compared to 19 out of 76 patients in the control group.

^◦^In the case group, information on TNM stage (8^th^ edition) was available for 9 patients, in the control group for 44 patients. In the case group, 3 out of 9 had a stage I tumor, compared to 15 out of 44 patients in the control group.
